# Beyond the plate: can a food-based science learning intervention improve preschool children’s fruit and vegetable consumption?

**DOI:** 10.1017/S1368980026101967

**Published:** 2026-01-23

**Authors:** Jocelyn B. Dixon, Elizabeth G. Blosser, Qiang Wu, L. Suzanne Goodell, Joseph L. Donaldson, Misty D. Lambert, Ryan Lundquist, Tammy D. Lee, Archana V. Hegde, Savannah Baldwin, Ashleigh Schmitt, Virginia C. Stage

**Affiliations:** 1 Department of Agricultural & Human Sciences, NC State University, NC State Extension, Ricks Hall, 1 Lampe Drive, Campus Box 7607, Raleigh, NC 27695-7607, USA; 2 Department of Public Health, East Carolina University, 115 Heart Drive, Greenville, NC 27834-4354, USA; 3 Department of Food, Bioprocessing and Nutrition Sciences, NC State University, Schaub Food Science Bldg, 400 Dan Allen Dr, Raleigh, NC 27606, USA; 4 Department of Mathematics, Science, & Instructional Technology Education, East Carolina University, 038 W.H. Smith Blvd, Suite 102, Greenville, NC 27834, USA; 5 Department of Human Development & Family Science, East Carolina University, 1400 Spring Garden Street, Greenville, NC 27858, USA; 6 NC Head Start, Early Head Start and Child Care Partnership, Telamon TRC, Raleigh, NC, USA

**Keywords:** Preschool, Head Start, Skin carotenoids, Food-based learning, Gardening

## Abstract

**Objective:**

To examine the efficacy of a food-based intervention on preschool children’s (3–5 years) fruit and vegetable (FV) consumption, as measured by skin carotenoid status (SCS) using the Veggie Meter^®^.

**Design:**

Quasi-experimental intervention with baseline (T1), pre-intervention (T2) and post-intervention (T3) assessments of children’s SCS. Intervention classrooms (ICs) received the programme, which featured food-based learning (FBL) and gardening. Comparison classrooms (CC) received a standard curriculum. Child and Adult Care Food Program (CACFP) aligned menus were identical across all centres. Intervention teachers participated in semi-structured interviews to contextualise quantitative findings.

**Setting:**

Head Start centres (*n* 7) across three counties in North Carolina.

**Participants:**

183 Head Start children (*n* 88 IC; *n* 95 CC)

**Results:**

During the intervention period (T2–T3), significant SCS increases were observed in both groups: IC (T2 = 253·7, sd = 77·7; T3 = 299·0, sd = 77·4) and CC (T2 = 226·6, sd = 77·5; T3 = 255·9, sd = 79·9). The IC demonstrated a greater gain in SCS (17·8 % gain) than the CC (12·9 % gain). However, additional analyses revealed no significant difference in the SCS rate of change over time (*P* = 0·33). Teachers reported that the intervention improved children’s willingness to try fruits and vegetables and encouraged positive feeding practices beyond the mealtime setting.

**Conclusions:**

The findings suggest that increased access to FVs through CACFP-supported meals and snacks may influence children’s overall improved FV consumption. However, improved food access paired with FBL may also support higher gains in FV consumption.

Dietary behaviours are shaped early in life, making preschool a critical period^(1)^. However, most children in the USA do not meet daily fruit and vegetable (FV) intake recommendations^(2)^, with children from households of limited resources consuming less FVs than their more affluent peers^(3)^. While children’s willingness to try foods may be influenced by developmental factors^(3)^, repeated exposure can increase acceptance of new or not-yet-preferred foods^(4,5)^ through direct interaction – tasting, touching, smelling or observing others^(3,6)^. Up to fifteen exposures may be needed to accept a new food, with more exposures potentially needed for familiar but not-yet-preferred foods^(3,4)^ or for neurodiverse children^(7,8)^. This number of exposures may be challenging for families facing food insecurity, as caregivers may be reluctant to repeatedly offer foods that children consistently reject^(3,9)^.

While the home plays a critical role in shaping eating habits, the early care and education (ECE) setting is often the first external setting to impact children’s health^(6)^. Children in full-time ECE consume 50–75 % of their daily intake away from home^(10)^, making ECE an essential setting for FV consumption. However, many policies assume that simply offering FVs will significantly improve children’s consumption. While this may work for some children, this approach overlooks the complex factors influencing children’s eating behaviours^(3,11)^. Preschool-aged children are undergoing cognitive developmental transitions and are still learning to interpret and respond to new sensory experiences, making appearance, texture and familiarity strong influences on their food choices^(3,12)^. Therefore, simply offering FVs is often not enough to ensure consumption.

Although educators value nutrition education, they lack time and face competing demands like ensuring children’s kindergarten readiness^(13–16)^. Teachers and administrators have indicated that integrating food and nutrition into other learning domains (e.g. math, science literacy) is a promising strategy to address these challenges^(11,14,15,17–19)^. This integrative approach, termed food-based learning (FBL), emphasises food exploration in non-pressuring settings, such as classroom activities or gardening, where children can interact with FV outside of mealtime and without the expectation to eat them. These contexts may be more effective than mealtime-only exposures, where pressure to eat can reduce children’s willingness to try foods^(6,11)^. Researchers have defined FBL as the ‘use of food as a tool to provide repeated exposure to improve children’s dietary behaviours and/or academic learning related to knowledge (e.g. science, mathematics and literacy) and/or skills (e.g. gross motor, fine, physical)’^(14)^. In action, FBL may look like exploring what makes broccoli green by steaming it and watching the water turn green to discuss the property of chlorophyll^(17)^; having children practise the mathematical concept of patterns by arranging tomatoes, spinach and cheese on a skewer^(19)^; reading a fiction or non-fiction book to children about FVs^(17)^; doing a FV puppet show or art collage^(20)^; or gardening activities related to the preparation, maintenance and/or harvesting of FV^(21)^. Importantly, FBL is an integrative approach and may be better aligned with the needs of teachers and administrators compared with traditional nutrition education, as it exposes children to healthy foods while simultaneously addressing the developmental and kindergarten readiness needs of young children^(18,19,21,22)^.

While studies have demonstrated that FBL can improve preschoolers’ preference and consumption of healthy foods^(11,17,19)^, more research is needed to assess its impact on intake using objective measures. Therefore, the purpose of our study is to examine the efficacy of a FBL intervention (*More PEAS Please!*) on Head Start children’s (3–5 years) FV consumption as measured by skin carotenoid status (SCS) using the Veggie Meter^®^. We hypothesise that children in the intervention group would demonstrate significant improvements in SCS compared with children in a comparison/delayed intervention group.

## Methods

### Study design

We conducted a mixed-methods quasi-experimental study during the 2023–2024 school year in partnership with one large Head Start programme serving three counties in central North Carolina. The Head Start programme assigned centres to either the intervention or the comparison/delayed intervention group based on the county. Children’s FV consumption was assessed quantitatively using SCS as measured by the Veggie Meter^®^^(23)^. Following the intervention, we conducted qualitative semi-structured interviews with participating teachers to contextualise child outcomes^(24)^.

### Participants and recruitment

We originally recruited 373 children enrolled in the partner Head Start programme. Children who did not speak English or had a disability or developmental delay that interfered with data collection were excluded. In total, we excluded 82 children from the study (*n* 16 intervention, *n* 66 comparison/delayed intervention) who met the exclusion criteria. We also excluded fifteen children who enrolled in the partner Head Start programme but never attended (*n* 10 intervention, *n* 5 comparison/delayed intervention). Lastly, we excluded an additional four children whose parents opted them out of the study (*n* 2 intervention, *n* 1 comparison/delayed intervention) or were not provided a waiver before enrolment (*n* 1 comparison/delayed intervention). A total of 272 children (*n* 125 intervention, *n* 147 comparison/delayed intervention) aged 3–5 years from twenty-eight classrooms (*n* 13 intervention, *n* 15 comparison/delayed intervention) across seven centres (*n* 4 intervention, *n* 3 comparison/delayed intervention) were eligible for participation. In the intervention group, trained teachers, staff and community partners collaborated to engage children in the FBL programme, *More PEAS Please!* (Preschool Education in Applied Sciences)^(25)^. In the comparison/delayed intervention group, children received the standard science curriculum (Teaching Strategies Gold^®^) and the intervention the following school year (2024–2025). To minimise dietary variability among children between centres, we partnered with a single Head Start programme where all centres followed the same 6-week cycle menu.

At the beginning of the school year (August–September), we informed families of enrolled children about the study through letters sent home and at least one centre-based parent meeting. Letters detailed the research and included a parental permission waiver. Only families opting their child out of the study needed to sign and return the waiver, either as a hard copy or electronically. Finally, we included lead or assistant Head Start teachers aged 18 years or older; thirty Head Start teachers were eligible to participate in the *More PEAS Please!* intervention. Thirty-eight teachers were allocated to the comparison/delayed intervention group and excluded from in-depth interviews, as they had not yet implemented the programme.

### Description of the More PEAS Please! programme

The *More PEAS Please!* programme is a multilevel intervention designed to improve preschool teachers’ science teaching practices while improving children’s FV consumption, science knowledge and language development^(26)^. Following a state-wide mixed-methods needs assessment^(14,16)^, we developed the programme with a transdisciplinary team of faculty, staff, Head Start teachers and community partners with expertise in ECE, teacher professional development, paediatric nutrition, nutrition education and early science education. We revised the final intervention using findings from our pilot test of the developed programme^(22,27)^. The final *More PEAS Please!* intervention consists of three integrated components: (1) teacher professional development, (2) FBL classroom learning for children and (3) centre-based environmental supports (e.g. one-on-one coaching, teacher-led learning communities (LCs) and science learning gardens).

Participating teachers were trained to implement *More PEAS Please!* through ongoing professional development, including attending a face-to-face 8-h Kick-Starter Workshop, completing eLearning modules, implementing classroom activities and engaging with environmental supports.

Teacher consent was obtained during the Kick-Starter Workshop held in October 2023. During this initial training, teachers participated in hands-on learning, met their PEAS Coach and Gardener and received materials, including a Teaching Guide and Science Teaching Kit. Following workshop training, teachers continued to receive professional development throughout the school year by completing six monthly eLearning modules and engaging in one-on-one coaching and centre-based LCs^(25)^. Teachers earned approximately thirty continuing education hours and up to $145 in gift cards as compensation.

Teachers implemented twelve hands-on science learning activities (four per month, February–April) featuring exploration of fresh FVs to support learning focused on the life sciences (e.g. living things, seeds, plants). The PEAS target FVs were selected for their familiarity, accessibility and cultural relevance within the partnering Head Start community^(28)^, including spinach, carrots, tomato, peas, broccoli, sweet potato, peppers, apple, mango and strawberries. Children also benefited from outdoor science learning gardens tied to classroom content. To support garden implementation, classrooms received portable raised beds and technical support from our Cooperative Extension-supported PEAS Gardener, who assisted with garden setup and led three gardening lessons focused on planting, caring for and harvesting vegetables (i.e. carrots, peas and radishes).

### Data collection and measures

We measured children’s SCS using the Veggie Meter^®^ and height and weight, a known covariate for SCS^(29)^, at three timepoints: baseline (T1, October), pre-intervention (T2, January/February) and post-intervention (T3, April/May). Data collection windows spanned 36–39 d (39 d at T1, 39 d at T2 and 36 d at T3), with standardised 59-d intervals between each wave. Children received a sticker for completing or attempting measurements. With parental awareness, we obtained child demographic information (e.g. birth date, race/ethnicity, sex, hearing or vision impairment, diagnosed or suspected disability or developmental delay) from school records. After the intervention (T3), we conducted teacher interviews to contextualise child outcomes; teachers received a $25 gift card as compensation for their time.

#### FV consumption (skin carotenoid status)

The Veggie Meter^®^ is a reflection spectroscopy (RS) device validated against plasma carotenoid concentrations to assess the concentration of carotenoids in the skin^(23)^. The device uses RS to non-invasively measure skin carotenoid concentrations, a biomarker of the past approximately 6 weeks of dietary intake of colourful fruits and vegetables (e.g. carrots, sweet potatoes, spinach)^(23,30–32)^. Veggie Meter^®^ scores range from 0 to 850^(23,33)^, with prior studies demonstrating positive correlations between proxy-reported FV intake and RS values in young children^(29)^. After setting up the Veggie Meter^®^, we allowed it to acclimate to the room conditions for at least 5 min before beginning assessments. Environmental conditions were not recorded; all data were collected indoors in low-humidity classrooms. Before measurement, children washed their hands with soap and water^(33)^. We used the single-scan mode to assess each child’s non-dominant ring finger (∼12 s per child) due to challenges with the three-scan method when working with young children, which requires multiple finger insertions^(29)^. We recalibrated the device hourly or after moving locations and used the same device across all time points for consistency^(33)^.

#### Height and weight (BMI percentiles)

We measured children’s weight to the nearest 0·10 kg using a digital body weight scale (Seca 869) and height to the nearest 0·10 cm using a portable stadiometer (Seca 213). We calculated BMI as weight divided by height squared (kg/m^2^) and derived BMI percentiles and z-scores using the U.S. Centers for Disease Control & Prevention’s age-and sex-specific growth charts, classifying children as underweight (< 5th percentile), normal weight (5th–85th percentile), overweight (85th–95th percentile) or obese (≥ 95th percentile).

#### Teacher interviews

A trained qualitative researcher, independent of programme delivery, conducted in-depth, semi-structured teacher interviews following the intervention implementation. To ensure trustworthiness, we trained the interviewer using the Goodell five-phase protocol, used a standardised interview guide, conducted field note-taking and recorded/transcribed each interview verbatim^(34,35)^. The interview guide focused on teachers’ implementation experiences and was pilot-tested with seventeen Head Start teachers to ensure clarity^(26,27)^. We audio-recorded and transcribed interviews using the Rev App on university-owned iPads. To protect identities, transcriptions and reporting use pseudonyms.

### Data analysis

We analysed quantitative data using SAS version 9.4 (SAS Institute Inc.). Children’s age (months), SCS and BMI variables were summarised using means (with sd) and compared between the two groups using two-sample *t* tests. Demographic characteristics were reported in frequencies and percentages and compared between the two groups using Fisher’s exact tests or *χ*^2^ tests. We summarised SCS and its changes from baseline to pre-intervention and then to post-intervention in means (sd) and compared the two groups with two-sample *t* tests. To further assess the impact of the *More PEAS Please!* intervention, we analysed SCS using a linear mixed model. Only cases with complete data on SCS at the three time points were included in the analysis. Little’s Missing Completely At Random test indicated missing completely at random; no significant differences were detected between the group of children included in analyses *v*. the group excluded (*P*-value > 0·32).

Our mixed linear model included demographic variables as covariates, a main effect for time, a main effect for group and a time-by-group interaction term. Head Start centres were treated as a random effect, and the model accounted for repeated observations on each child using a first-degree heterogeneous autoregressive error structure. The Kenward–Roger method was used to estimate df, and model assumptions were checked with residual Q–Q plots. We set our significance level at *P* ≤ 0·05. Finally, a *post hoc* power analysis revealed that a sample of 183 children (sample size of complete cases) among seven centres (four intervention and three comparison/delayed intervention/delayed intervention) would provide 80 % power to detect an effect size about 0·9–1·3 (Cohen’s d) between the two treatments arms with a significance level of 0·05 using a two-sided two-sample *t* test when the intraclass correlation coefficient within the centres is assumed to be 0·1.

To analyse qualitative data, we used phenomenological methods, drawing on van Manen and Interpretive Phenomenological Analysis, to better understand teachers’ lived experiences implementing PEAS and contextualise their perception of its influence on children’s dietary outcomes^(24,36)^. To begin, two trained analysts independently read all transcripts twice. Coders then began preliminary independent coding and highlighting key concepts. Analysts met three times weekly, collectively reviewing each transcript, and began compiling a coding template. Analysts then compared assigned codes, line by line, in each manuscript until 100 % consensus was reached. In the case of disagreement, a third author served as the tiebreaker. Analysts continually developed and refined the codebook, which defined codes and outlined inclusion/exclusion criteria throughout this process.

Additionally, analysts documented memos, potential themes and questions throughout this process, which serve as additional documentation of the coding process throughout data analysis. Analysts then organised codes into larger categories to derive final themes from the data. Final themes were elicited through horizontalisation, where analysts described significant participant experiences through textural (‘what’) and structural (‘how’) descriptions for the whole dataset^(24)^. This article only reports themes specific to the research question focused on FV consumption. To maintain trustworthiness, analysts held regular team meetings to code, debrief, confirm data saturation and refine themes^(35)^.

## Results

### Sample characteristics

A total of 183 children were included in the analysis (*n* 88 intervention, *n* 95 comparison/delayed intervention). At baseline, the combined sample of children had an average age of 4·11 (±0·57). The sample was predominantly female (53 %), Black/African American (57·4 %) and non-Hispanic (73·2 %) (Table 1). At baseline, we observed significant differences in children’s age (*P* = 0·004), race (*P* < 0·001) and ethnicity (*P* = 0·03) between the two groups. BMI percentiles were not significantly different (*P* = 0·15) between groups, but BMI categories were (*P* = 0·01). At baseline, the mean BMI z-score for the sample was 0·66 (sd = 1·19), with no significant differences between intervention and comparison groups. In general, children in our intervention group were slightly older and more likely to be Black/African American and non-Hispanic, have a lower BMI percentile and have more frequently reported social-emotional concerns. Overall attrition for SCS was 29·6 % for the intervention group and 35·4 % for the comparison group. Attrition analysis revealed that between children who completed all SCS data collection time points and those who did not, there was no significant difference. Of the larger teacher sample (*n* 24), a total of nineteen teachers participated in post-intervention interviews. The interviewed teachers had an average age of 41·38 years (sd = 12·70), were all female and were predominantly Black/African American (84·20 %) (Table 2).

**Table 1.** Demographics at baseline for children in intervention and comparison groups (*n* 183)

	Intervention group (*n* 88)		Comparison group (*n* 95)			Cases with missing SCS data (*n* 89)		
Characteristics	Mean	sd	Mean	sd	*P*-value*	Mean	sd	*P*-value**
Age (months)	50·8	6·1	48·0	7·2	0·004^†^	49·9	6·5	0·49^†^
Age (years)	4·2	0·51	4·0	0·60	0·004^†^	4·2	0·57	0·49^†^
BMI percentile	63·6	27·3	69·6	29·1	0·15^†^	64·3	28·9	0·52^†^
	*n*	%	*n*	%	*P*-value*	*n*	%	*P*-value**
Sex					0·55^‡^			0·70^‡^
Male	39	44·3	47	49·5		39	43·8	
Female	49	55·7	48	50·5		50	56·2	
Race					< 0·001^‡,||^			0·51^‡,||^
Black/African American	62	70·5	43	45·3		45	50·6	
White	4	4·6	23	24·2		17	19·1	
Indian Alaskan	0		0			2	2·2	
Pacific Islander	0		0			1	1·1	
Multi/bi-racial	8	9·1	11	11·6		10	11·2	
Other	14	15·9	17	17·9		10	11·2	
Missing	0		1	1·1		4	4·5	
Ethnicity					0·03^‡^			0·88^‡^
Non-Hispanic/Latino	71	80·7	63	66·3		61	71·8	
Hispanic/Latino	17	19·3	32	33·7		24	28·2	
BMI category					0·01^§^			1·0^§^
Underweight	2	2·3	2	2·1		2	2·4	
Normal weight	58	65·9	51	53·7		51	60·0	
Overweight	21	23·9	15	15·8		17	20·0	
Obese	7	8·0	27	28·4		15	17·7	
Developmental concerns^¶^								
Hearing	4	4·7	1	1·1	0·19^‡^	2	3·1	1·00^‡^
Vision	17	19·5	14	14·9	0·44^‡^	7	10·9	0·32^‡^
Developmental	19	21·6	24	14·7	0·25^‡^	12	18·5	1·00^‡^
Social-emotional	12	13·6	4	4·2	0·03^‡^	3	4·6	0·42 ^‡^

SCS, skin carotenoid scores.

Significance level at </= 0·05.

* Comparing intervention and comparison groups.

** Comparing complete cases and cases with missing SCS.

† Two-sample *t* test; ‡Fisher’s exact test; §*χ*^2^ test; ||comparing % Black/African American students.

¶ Developmental concerns were noted in the children’s charts as identified during Head Start’s beginning-of-year child screenings and assessments. According to the Program Performance Standards, programmes are required to complete or obtain existing records of current developmental screenings to identify concerns in the areas of development, behaviour, motor, language, social, cognitive and emotional skills within 45 d of when a child first attends the programme (1302·33 Child Screenings and Assessments).

**Table 2:** Demographics at baseline for interviewed total teachers in intervention group (*n* 23) *v*. intervention teachers interviewed (*n* 19)

Characteristics	All intervention teachers (*n* 23)				Interviewed teachers only (*n* 19)			
	*n*	%	M	sd	*n*	%	M	sd
Age	–		40·29	11·90	–		41·38	12·70
Sex								
Male	0	0	–		0	0	–	
Female	23	100	–		19	100	–	
Race								
Black/African American	19	82·6	–		16	84·2	–	
Hispanic	2	8·7			2	10·5		
White	1	4·3	–		1	5·3	–	
Asian	1	4·3			0	0		
Ethnicity								
Non-Hispanic/Latino	20	87·0	–		16	84·2	–	
Hispanic/Latino	2	8·7	–		2	10·5	–	
Prefer not to answer	1	4·3	–		1	5·3	–	
Education								
Master’s degree or some graduate-level work	4	17·4	–		4	21·05	–	
Bachelor’s degree	11	47·8	–		8	42·1	–	
Associate’s degree	4	17·4	–		4	21·05	–	
Other	4	17·4	–		3	15·8	–	
Years working in current head start programme	–		3·35	4·77	–		3·20	3·93

### FV consumption (skin carotenoid status)

**Figure 1.**
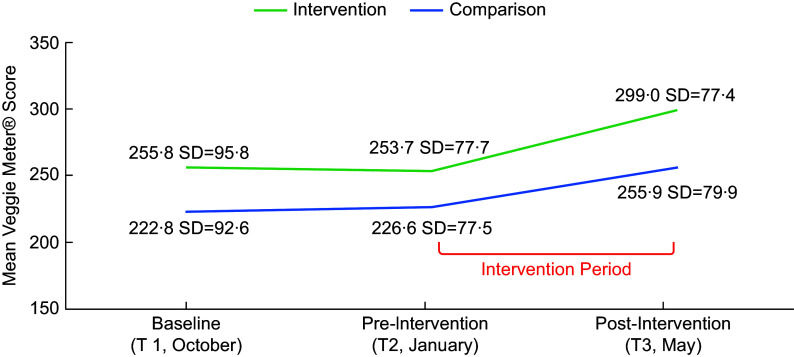
Skin carotenoid status (SCS) as measured by the Veggie Meter^®^ at baseline (T1), pre-intervention (T2) and post-intervention (T3). The intervention group showed an improvement of 17·8 % during the intervention period (T2–T3), while the comparison group improved by 12·9 %, both statistically significant (*P* < 0·001). The linear mixed model did not reveal a significant interaction between the two intervention groups and time (*P* = 0·33); both groups showed significant improvements over time (*P* < 0·001). Model covariates included age (months), sex, BMI percentile, race/ethnicity (Black/African American, Hispanic) and developmental concerns (i.e. vision, development and social-emotional concerns).

Figure 1 displays mean SCS scores over time as measured by the Veggie Meter^®^ (complete cases included *n* 88 intervention, *n* 95 comparison/delayed intervention). The intervention group had a significantly higher mean SCS than the comparison/delayed intervention group at baseline (T1) (255·8 (95·8) *v*. 222·8 (92·6), *P* = 0·02), pre-intervention (T2) (253·7 (77·7) *v*. 226·6 (77·5), *P* = 0·02) and post-intervention (T3) (299·0 (77·4) *v*. 255·9 (79·9), *P* < 0·001). The intervention group had a similar mean baseline to pre-intervention change (intervention M = –2·0, sd = 90·4; comparison/delayed intervention M = 3·8, sd = 77·8, *P* = 0·64), but a non-significantly higher mean pre-intervention to post-intervention change (intervention M = 45·3, sd = 68·1; comparison/delayed intervention M = 29·3, sd = 70·9, *P* = 0·12) than the comparison/delayed intervention group. The intervention group demonstrated a 17·8 % improvement in FV consumption pre-intervention to post-intervention, compared with 12·9 % in the comparison/delayed intervention group, both statistically significant (*P* < 0·001).

The linear mixed model analysis used age (months), sex, BMI percentile, race/ethnicity (Black/African American, Hispanic) and developmental concerns (i.e. vision, development and social-emotional concerns) as covariates. While BMI percentiles were not significantly different between groups, it has been previously cited as a significant covariate in SCS analyses^(30)^. The mixed model analysis of SCS revealed no statistically significant intervention group × time interaction, suggesting no differing effect of the *More PEAS Please!* intervention as compared with the comparison/delayed intervention group. Both groups showed significant improvements over time (*P* < 0·001), but they showed no significant differences in mean baseline (T1) to pre-intervention (T2) change (d = –6·7, 95 % CI = (–30·8, 17·4), *P* = 0·58) and in mean pre-intervention (T2) to post-intervention (T3) change (d = 15·7, 95 % CI = (–5·4, 36·9), *P* = 0·15)) (Fig. 1, Table 3). The mixed model residual demonstrated a close to normal distribution in a normal Q–Q plot, justifying the chosen model.

**Table 3:** Mixed model effects and *P*-values for children’s skin carotenoid status as measured by the Veggie Meter^®^ (*n* 183)

Model effect	Veggie Meter Score^®^		*P*-value
	B	95 % CI	
Intercept	315	233, 397	
Age (months)	−0·31	–1·83, 1·22	0·693
BMI percentile	−0·53	–0·88, –0·17	0·004
Sex (Female *v*. Male)	−2·46	–23·0, 18·1	0·814
Race (Other *v*. Black)	−2·69	–29·7, 24·3	0·844
Ethnicity (Non-Hispanic *v*. Hispanic)	−8·23	–37·0, 21·0	0·580
Vision concerns (Yes *v*. No)	−10·7	–37·0, 15·7	0·425
Developmental concerns (Yes *v*. No)	11·8	–18·8, 42·4	0·447
Social-emotional concerns (Yes *v*. No)	13·8	–27·7, 55·4	0·512
Time			
T1 *v*. T3	−33·7	–54·3, –13·1	< 0·001
T2 *v*. T3	−29·7	–44·4, –15·0	
Intervention group (Intervention *v*. Comparison)	39·1	15·2, 63·1	0·005
Time × Intervention group			
T1 *v*. T3 × Intervention *v*. Comparison	−9·02	–38·8, 20·7	0·325
T2 *v*. T3 × Intervention *v*. Comparison	−15·7	–36·9, 5·44	

T1 = baseline; T2 = pre-intervention; T3 = post-intervention. Analysis only used cases for which complete data were available (Intervention *n* 88; Comparison *n* 95).

### Teacher in-depth interviews

Three emergent themes related to children’s FV consumption were identified: *improving children’s willingness to try through food-based learning*, *teachers’ feeding practices during food-based learning* and *teachers’ motivations to improve diet quality* (Table 4).

**Table 4:** Themes, subthemes and representative quotes: Head Start teachers’ perceptions of the *More PEAS Please!* effect on children’s fruit and vegetable consumption (*n* 19)

Theme	Subtheme	Representative quote
Improving children’s willingness to try through food-based learning	Food-based science activities	Changing Children’s Willingness to Try: ‘I was real shocked. I think from us talking about ‘em so much and learning about roots vegetables and this kind of vegetable is from a stem and this vegetable is from a tree… and categorizing ‘em and then learning to plant in ‘em and reading about them, I think it made them actually want to try them more because when we had those food experiences, I was so surprised at some of the children who was trying those different foods. I was real surprised’. (Faith) Engaging Children in Hands-on Learning: ‘When we started with the seeds [unit], immediately they were looking for seeds in their lunch food or in their snacks, and they wanted to get the handheld magnifying glasses or draw a picture of it… they were outside looking for seeds and wanted to bring them in and sort them or decide what kind they were. I mean, it was amazing. They came up with things that we didn’t think of. Even to this day when we’re eating a vegetable or something that they find seeds, they’re all peeling their food apart, looking for the seeds… they were making connections with just about everything we talked about within the PEAS program’. (Aaliyah)
	Kids cooking activities	Changing Children’s Willingness to Try: ‘We mixed the yogurt, the honey and the cinnamon and everything. And I think that was the kids’ favorite one because they could not get enough of that yogurt dip. They loved it. And even with the vegetables that they ate with it, I mean, they were trying the vegetables just to eat the yogurt dip too…And it’s probably something that they wouldn’t try at home, but they tried it at school because they were involved in making it and everything. So that’s what made it fun for me’. (Jesse) Engaging Families: ‘My parents said, “I never knew my child would even attempt to try these things”. Today, the parents at home are afraid to allow their child to use a kid-friendly knife, but at school they were using it. And so then the children started going home and they were talking about the experiences in the classroom. And so the parents started allowing the child to help at home, like prepping dinner things’. (Camille)
	Garden-based activities	Changing Children’s Willingness to Try: ‘But I think my biggest one was the garden where they was able to go outside and watch and see what they’re growing and just being a part of that. And also being able to eat it. Because normally when kids, they grow something, they don’t really see the finished product of it, it growing. So that was pretty much my favorite part of the whole PEAS’. (Izabel) Classroom to Garden to Table: ‘So when we were talking about the plants and saying, “oh, they come from seeds”, a few kids were like, “oh, we’re talking about favorite foods”…And we were like, “that comes from the seed, that’s how they grow”. So we were able to bring that in and even when they talk about things now we’re like, that comes from the ground. “We grow those too”, or we were able to apply, “oh hey, we have the garden. That’s where they come from”’. (Blaire)
Teachers’ feeding practices during food-based learning	Supportive practices	Role Modelling: ‘I had to look at it myself and say, “okay, you got to model this for the kids”. So to even try it myself, I wasn’t used to eating snacks like that. And then just talking to them about how those vegetables are good for our bodies’. (Sarah) ‘I used those same techniques [I learned at the PEAS Kick-Starter Workshop] when I saw my students weren’t trying certain things they had ever been exposed to, like the radish. And then I was like, just taste it, or smell it’ (Krystal). Another teacher shared, ‘they observed it, they described what they saw, colors of it, the texture, smelling it’. (Sarah) Respecting Children’s Preferences and Autonomy: ‘We would just say, if you like it, give us a thumbs up. If you didn’t like it, give it a thumbs down. And then we just kind of left it like that. We didn’t go into more detail because we didn’t want to have another child shy another child away from something that they liked’. (Kamara)
	Unsupportive practices	Dichotomising Foods: ‘Some of them really liked it and some of them didn’t. We talked about how it was healthy and how the vegetables, I would say, show them a picture of a cake. “Is this healthy?” “No, no, it’s not healthy”. I said, “it’s good, but it’s not healthy”’. (Mavis) Forcing Children to Try Foods or ‘Clean Their Plate’: ‘I said try it, you might like it. Some of them did fight back on me, but other ones didn’t. I always tell my kids, don’t say you don’t like it if you never tried it. Always try something. Some of the kids didn’t really care for it, but I’m always going to try to get them to eat everything that comes on their plate’. (Riley)
Teachers’ motivations to improve diet quality	Professional motivations	Supporting Children’s Health: ‘…to tell the children that the best thing for their bodies, for their growth is to eat foods and vegetables and healthy food’. (Sophia) Sharing with Families: ‘We took one of the recipes from the PEAS book and allowed the families to create it… I had parents coming back and asking me could I send them the copy of the recipe. They was like, I never thought about putting these ingredients together’. (Camille)
	Personal motivations	Improving Personal Health: ‘I wasn’t big on fruits and being healthy, but after PEAS…I probably only eat out once or twice a week and I have been packing my lunch and I’ve been eating fruits, snacking on fruits instead of candy…Just being better. I cut back on my kids junk food…Now when I go to the store, I get bananas, apples, applesauce…I just changed how we was eating’. (Anita)

#### Theme 1: Improving children’s willingness to try through food-based learning

Teachers expressed that PEAS’ FBL activities caused a positive shift in children’s willingness to try new and not-yet-preferred foods. Teachers identified three key FBL settings: *Food-Based Science Activities*, *Kids Cooking Activities* and *Garden-Based Activities*. While each setting is distinct, teachers felt that each positively impacted children’s willingness to try foods.

Teachers described *Food-Based Science Activities* in various ways, often highlighting their improvement of children’s mealtime behaviours, including an increase in children’s curiosity during activities outside of mealtime, which frequently translated into increased engagement during mealtimes. Faith described that before PEAS, children ‘don’t try a lot of foods. A lot of times our lunch lasts 20 min because nobody really eats’. Teachers shared that the programme not only increased children’s willingness to try foods but also exposed them to new ones: ‘I’m sure if it wasn’t for PEAS, a lot of those experiences that they would’ve never had, they would’ve never tasted a radish’ (Krystal). Reflecting on the shift in behaviour, Krystal added, ‘[it’s] a wild moment when the kids started being more interested in healthy foods. And even when we would have our lunch, they would go back and use that talk about asking, ‘Where did this pea come from?’

Teachers also emphasised that the impact of *Kids Cooking Activities*, such as mixing, cutting with child-friendly knives, measuring and assembling PEAS recipes, improved children’s willingness to try foods. They noted that children enjoyed the activities, felt proud of their work and valued the teamwork it required: ‘It was like they were so proud of themselves because we had made something as a group and were eating it together as a family’ (Logan). Many noted how children especially loved using the child-friendly knives and that the successes of classroom cooking led some teachers to share these experiences with families. Lastly, teachers described *Garden-Based Activitie*s as increasing children’s willingness to try foods. During garden-based activities, children applied their classroom knowledge outdoors. Teachers often overheard children making connections between PEAS content and daily experiences. Overall, teachers agreed that gardens provided sensory exposure to FVs, making children more open to trying them. Most teachers felt the gardens were children’s favourite part of PEAS.

#### Theme 2: Teachers’ feeding practices during food-based learning

Teachers described *supportive practices* and *unsupportive practices* for engaging children with healthy foods during PEAS. *Supportive practices* included role modelling, sensory exploration and respecting children’s preferences and autonomy. Teachers role modelled eating healthy foods themselves, while others encouraged sensory exploration beyond the sense of taste. Teachers respected children’s autonomy by not forcing them to try or like anything. For example, one teacher used a thumbs-up system in her classroom to allow children to express their preferences for a food while respecting the preferences of their peers. Another teacher taught polite phrases for expressing dislikes, ‘So we wouldn’t down talk anybody else, whoever is trying it. I know one person said, “Oh, I don’t like it”. Now everyone’s thinking “I don’t like it”. So trying to rephrase it and they said, “Oh, this is interesting”. The other person is now thinking “Oh, it’s good”. And she said, “Oh yeah, I like this. This is interesting too”’ (Luna).

Only two teachers described *unsupportive practices* that are inconsistent with evidence-based best practices. One teacher described labelling foods to children as ‘good/healthy’ or ‘bad/unhealthy’ in attempts to get children more interested in trying healthy foods. Another teacher tried forcing children to try foods, or making children eat everything served: ‘everybody would have to taste it, even if we didn’t want to’ (Mavis).

#### Theme 3: Teachers’ motivations to improve children’s diet quality

Teachers were motivated during PEAS through both *professional* and *personal motivations*. Teachers felt that preparing children for healthy futures was a part of their role as ECE educators. They expressed concern that families have replaced fast food with nutritious meals, describing it as ‘so much cheaper than buying fresh fruit and vegetables’ and emphasising that ‘most parents are living a busy lifestyle, just don’t slow down enough to cook. It’s much easier to just pop through a drive-thru’ (Logan). Other teachers felt concerned that families may be facing food insecurity at home. In cases where children were not be as willing to eat foods in PEAS, teachers were concerned that children may not be eating adequate food before going home: ‘So when they’re not eating the snack, because we’re making our own ranch and eating vegetables out the garden, even though it’s good to get them to try, it just was like, dang, they’re not eating’ (Nikki). In response to these concerns, teachers described how they tried to engage families through informal conversations or by inviting families to try PEAS recipes in the classrooms with children. Finally, teachers reported that PEAS influenced their personal eating habits by becoming more mindful of the meals they make for their own family. Though the extent of these changes was not measured, teachers noted making positive shifts alongside the children in their classrooms.

## Discussion

This study examined the efficacy of the *More PEAS Please!* intervention on Head Start children’s (3–5 years) FV consumption using SCS measured by the Veggie Meter^®^. We hypothesised greater SCS improvements in the intervention group; however, both groups experienced improvements. Interviewed teachers provided additional insight into children’s engagement with new and not-yet-preferred foods inside and outside the meal and snack time environment throughout PEAS. The positive trends in FV consumption combined with teachers’ positive experiences highlight the promise of integrated, experiential, food-based science learning in early childhood settings.

From pre-to post-intervention, both intervention and comparison/delayed intervention groups experienced significant gains in SCS. One potential explanation for non-significant differences between groups is the timing of data collection. Our partner followed the traditional academic calendar (August–May), restricting our ability to measure children’s SCS during the summer. Thus, our post-intervention data were collected in May, likely reflecting FV consumption from March. While prior controlled feeding studies demonstrate that SCS can change within 2–4 weeks^(17,30)^, those interventions involved prescribed, consistent carotenoid intake. Plasma carotenoids respond quickly to dietary changes; however, carotenoids accumulating in the skin are released more slowly and may take up to 8 weeks to accumulate.^(23,30,32)^ Because our study relied on a community-based design and measured skin carotenoids based on children’s self-directed behaviour change (rather than prescribed intake), a longer observation window may be required to capture cumulative changes in SCS. Measuring SCS in July, which reflects dietary intake from May, would have yielded a more accurate depiction of the full intervention effect. Future studies may consider implementing interventions in year-round facilities to allow for a longer measurement window, or including a plate-waste assessment in addition to SCS to assess short-term effects within programmes following a traditional school calendar.

While children have reliable access to FVs at school, research suggests that this access may not extend to home^(37)^, making school-based access especially critical. In our study, both groups had improved diet quality, which we suspect is related to increased access to FVs through meals and snacks supported through Head Start’s federal mandate to participate in the Child and Adult Care Food Program (CACFP)^(38)^. A similar increase in both intervention and comparison/delayed intervention groups has been observed in other studies with similar conclusions^(11)^, underscoring the protective role of programmes like CACFP against food insecurity and low diet quality among at-risk children. Although our study did not observe a decline in SCS over winter break, a prior study conducted in a rural NC-based Head Start observed a significant drop when children were not in school, likely due to this ‘access gap’ and potential higher risk for experiencing food insecurity when not in school^(17)^. However, qualitative data from our study suggest that some families still rely on fast food due to convenience and cost, pointing to the need for increased family education and engagement, which ultimately care for children in the long term.

In our study, both groups experienced significant SCS increases, with the intervention group’s larger gain (17·8 % *v*. 12·9 %), suggesting a potentially meaningful impact. Unlike the comparison/delayed intervention group, the intervention group’s change exceeded the Veggie Meter^®^’s theorised average margin of error of 14 % calculated in a multi-state study across eight sites using the CV (sd divided by the mean of five repeated scans per participant)^(39)^. Prior research suggests that access to FVs, familiar or unfamiliar, does not guarantee consumption^(11,40)^. Increasing young children’s willingness to try foods requires repeated exposures using multi-sensory engagement (e.g. smelling, touching, tasting)^(6)^. It may take 8–15 exposures for a child to accept a new food^(4,41)^ and even more exposures for a familiar food^(3,4)^, so combining hands-on, active learning approaches using food (e.g. growing, tasting, preparing) *with access to healthy foods* is an effective approach for improving young children’s FV consumption^(6,9,11)^. These findings highlight the importance of continued support for Head Start’s efforts to improve children’s food and nutrition education and access.

Teacher insights contextualised changes in FV consumption among intervention group children. Teachers perceived PEAS positively influenced child health behaviours by increasing children’s willingness to try foods through hands-on food-based science, cooking and gardening activities. Teachers described specific strategies such as role modelling^(14,42,43)^ and encouraging sensory exploration *beyond the sense of taste* (e.g. sight, smell, touch, hearing). Although a few FBL interventions promote multi-sensory food exploration outside mealtime^(11,17,19–21)^, to our knowledge, this study is the first to report teachers’ direct accounts of these practices. These strategies align with Johnson’s two-stage model, which emphasises the importance of observational learning and repeated sensory engagement^(3)^.

A few teachers reported using unsupportive practices such as pressuring children to eat foods served to them^(10,44)^. Head Start teachers often know that the families they serve may face food insecurity^(37)^. Additionally, many Head Start teachers come from limited-resource backgrounds themselves and may face similar challenges with food insecurity^(45)^. Thus, some teachers may adopt unsupportive feeding practices, such as pressuring children to eat, due to the worry that children may not have adequate food at home^(46)^. Additional training in the application of supportive feeding practices^(10,14,15,44)^ outside of the mealtime, as well as additional research on the contextual factors that influence early childhood educator behaviours^(47,48)^, should be explored.

### Limitations

This study involved a small sample from a single Head Start programme in the southeastern USA, limiting generalisability. Additionally, group assignment followed the community partner’s preference of assignment by county, rather than randomisation, introducing potential selection bias, despite statistical adjustment for baseline differences. While randomised controlled trials are often considered the gold standard, they can present challenges for community-based work, including limited external validity and ethical or practical concerns when community autonomy is restricted. In this study, we prioritised honouring our Head Start partners’ preferences in order to promote shared decision-making, strengthen trust and support authentic collaboration^(49)^. The absence of a significant group-by-time interaction may be partially attributed to these differences. Future studies should consider a randomised controlled design and account for constraints such as Head Start’s traditional calendar, which required earlier-than-ideal post-intervention SCS measurements^(17,30)^. Additionally, since the Veggie Meter^®^ does not detect colourless carotenoids or other phytochemicals in commonly consumed healthy foods like apples or bananas, future studies may consider incorporating plate-waste assessments to capture broader FV intake. Nevertheless, RS remains the best objective measurement of young children’s FV intake in a community setting^(30)^. Finally, only intervention teachers were interviewed; including comparison/delayed intervention teachers in future studies could provide insight into how perceptions differ by programme exposure.

### Conclusion

Findings revealed improved FV consumption in both groups, suggesting that increased access to healthy foods through CACFP-supported meals may have positively impacted outcomes. While NS, the intervention group experienced greater SCS gains (approximately 5 %), indicating that FBL, paired with improved food access, may enhance outcomes beyond access alone^(11,50)^. Future research should compare FBL programmes with and without CACFP to explore the effect of access within ECE settings. Finally, these findings justify the Office of Head Start’s continued efforts to support nutrition education and food access through Program Performance Standards and underline the critical importance of these public health strategies for long-term child and family health.
